# Automatic Cow Location Tracking System Using Ear Tag Visual Analysis

**DOI:** 10.3390/s20123564

**Published:** 2020-06-23

**Authors:** Thi Thi Zin, Moe Zet Pwint, Pann Thinzar Seint, Shin Thant, Shuhei Misawa, Kosuke Sumi, Kyohiro Yoshida

**Affiliations:** 1 Graduate School of Engineering, University of Miyazaki, Miyazaki 889-2192, Japan; smithmoezet01@gmail.com (M.Z.P.); pannthinzarseint@gmail.com (P.T.S.); shinthant3010@gmail.com (S.T.); hl15044@student.miyazaki-u.ac.jp (S.M.); 2 Interdisciplinary Graduate School of Agriculture and Engineering, University of Miyazaki, Miyazaki 889-2192, Japan; hc11093@student.miyazaki-u.ac.jp; 3 Advanced Telecommunications Research Institute International, Kyoto 619-0237, Japan; yoshida-kyohiro@atr.jp

**Keywords:** object detector, ear tag recognition, digit segmentation, convolutional neural network, location searching

## Abstract

Nowadays, for numerous reasons, smart farming systems focus on the use of image processing technologies and 5G communications. In this paper, we propose a tracking system for individual cows using an ear tag visual analysis. By using ear tags, the farmers can track specific data for individual cows such as body condition score, genetic abnormalities, etc. Specifically, a four-digit identification number is used, so that a farm can accommodate up to 9999 cows. In our proposed system, we develop an individual cow tracker to provide effective management with real-time upgrading enforcement. For this purpose, head detection is first carried out to determine the cow’s position in its related camera view. The head detection process incorporates an object detector called You Only Look Once (YOLO) and is then followed by ear tag detection. The steps involved in ear tag recognition are (1) finding the four-digit area, (2) digit segmentation using an image processing technique, and (3) ear tag recognition using a convolutional neural network (CNN) classifier. Finally, a location searching system for an individual cow is established by entering the ID numbers through the application’s user interface. The proposed searching system was confirmed by performing real-time experiments at a feeding station on a farm at Hokkaido prefecture, Japan. In combination with our decision-making process, the proposed system achieved an accuracy of 100% for head detection, and 92.5% for ear tag digit recognition. The results of using our system are very promising in terms of effectiveness.

## 1. Introduction

**Figure 1:**
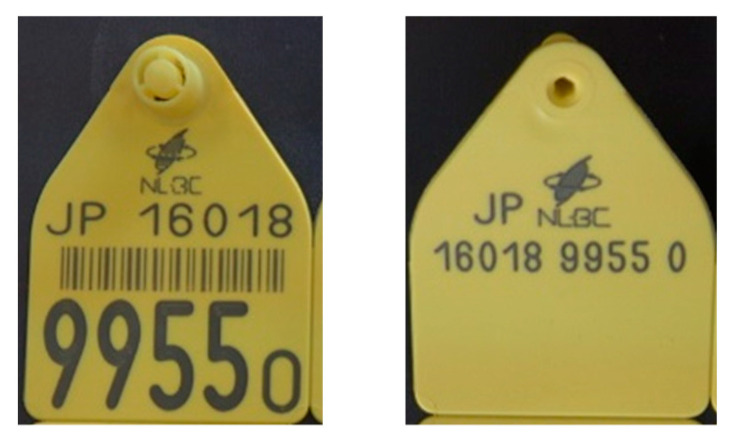
A sample ear tag that is attached to the cow’s ear in Japan.

Smart dairy farming emerged from the concept of Precision Agriculture, in which IoT technologies and artificial intelligence analysis are put to efficient use. Using these technologies to provide individual care for cows is fundamental to the future of dairy farming. Most dairy farms around the globe adhere to international ISO standards in identifying individual cows. At birth, every calf gets a unique ID number. This number is registered in a database, e.g., the database in the National Livestock Breeding Centre (NLBC) of Japan, as described in [1]. An example of an ear tag image is given in Figure 1.

This ID number is composed of the responsible organization in a country (NLBC in Japan), the country code (JP), and the complete unique life number 16018 9955 0, in digits and as a barcode. The last four digits (9955) are highlighted as the work number which is most commonly used by the farmer. Every cow on a dairy farm has a unique number. Some other organizations also link this unique number to an electronic number. They make a unique RFID (Radio Frequency Identification Device) transponder that can be used in combination with one of the yellow ear tags.

The principle behind electronically readable RFID tags is the same as that used for RFID in other official identification and registration schemes. These tags can improve cow identification, but the long-term irritation of wearing them can also induce stress on cows. Using RFID tags throughout a farm can be expensive, so other useful and cost-effective systems are needed for the automatic monitoring of cows.

In recent years, researchers have been combining computer vision techniques with machine learning, achieving much success in many areas. Using a computer vision-based system that is specifically designed for cow management can reduce costs, especially by reducing the labor involved in monitoring each individual cow. In this paper, we propose an individual cow identification system using ear tags, expecting that this system will make important contributions to the implementation of precision dairy farming.

This paper is organized as follows. In Section 2, we review the literature, noting related research. We describe our proposed methods in Section 3. The experimental results are shown in Section 4 and Section 5 provides the conclusions.

## 2. Related Works

In this section, we review some related research, specifically that concerning cow identification and monitoring. The black and white pattern on a cow’s body is used for identification and classification, using image processing techniques combined with deep learning [2]. Image processing techniques are used to extract the cow body region and extracted cow body images are subsequently applied to deep learning for identification. An identification system is needed to monitor a cow’s health and status for dairy farms. By using a cow’s ear tag, the authors proposed an individual cow identification system to be applied in precision dairy farming [3]. In [4], the authors proposed an individual cattle identification system using a convolutional neural network (CNN), and a long short-term memory (LSTM) network. Due to the complex layout of dairy farms, it is sometimes difficult to detect and track cows. A custom filter layer combined with YOLO v3 [5] to detect key parts of cows is proposed in [6] to overcome these problems. References [7,8,9] encompass a framework for detecting and recognizing the faces of cows. Faster R-CNN in [7] concerns cow face detection, and PNASNet-5 as described in [8] is applied to detection and recognition in [9]. As documented here, machine learning and deep learning techniques are finding applications with promising results.

To extract prominent features, the authors in [10] used muzzle print images and two detectors: Speeded-up robust features (SURF) and maximally stable extremal regions (MSER). These extracted features are finally combined into a bag-of-visual-words (BoVW) model for classification. The color threshold method is used for detecting ear tags, followed by applying a flood fill algorithm, and a Hough transform and projection method for segmenting ear tags, as proposed in [11]. For classifying ear tag digits, the authors used the methods of k-nearest neighbor and support vector machines. Nowadays, the usage of machine learning and deep learning techniques are widely used in different areas of fields [12,13]. Character segmentation is performed using a hybrid binarization technique in paper [14]. In this paper, license plates are localized based on texture features and rotated using the Hough transform. The authors in [15] apply the principle components analysis (PCA) to extract digit features and the backward propagation neural network to classify features. For invariance by rotation, scales and translation of digits, the authors in [16] use fractal dimension, lacunarity, and Hu’s moments as features. To recognize text on low-resolution images, the authors in [17] use a character recognizer and word recognizer. They use the convolutional neural network (CNN) as the character recognizer. The language-neutral model and dictionary model are used as word recognizers in their system. For identifying cows, features such as body parts, ear tags, and the head area are used, though these methods are subject to ongoing research.

## 3. Proposed System

**Figure 2:**
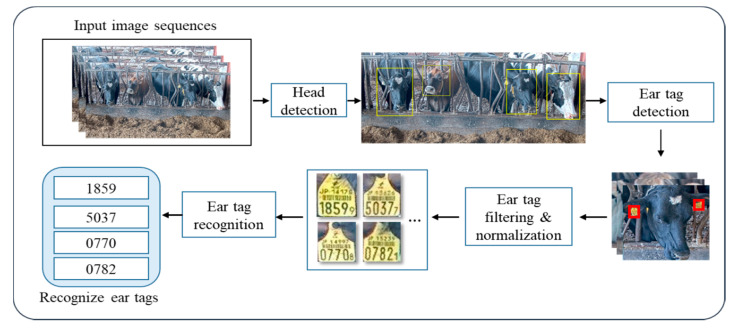
The architecture of the proposed system.

Our proposed system aims at obtaining an intelligent cow tracking system by using ear tag information. The experimental environment is composed of a feeding station under the monitoring of the front camera. The proposed system is mainly composed of five parts: (1) Cow head detection and localization, (2) ear tag detection and filtering, (3) normalization for ear tag alignment, (4) ear tag recognition process, and (5) decision making, which involve list pairs of the related camera ID and ear tag ID to provide the search system. The overall system architecture is shown in Figure 2.

### 3.1. Cow Head Detection and Localization

**Figure 3:**
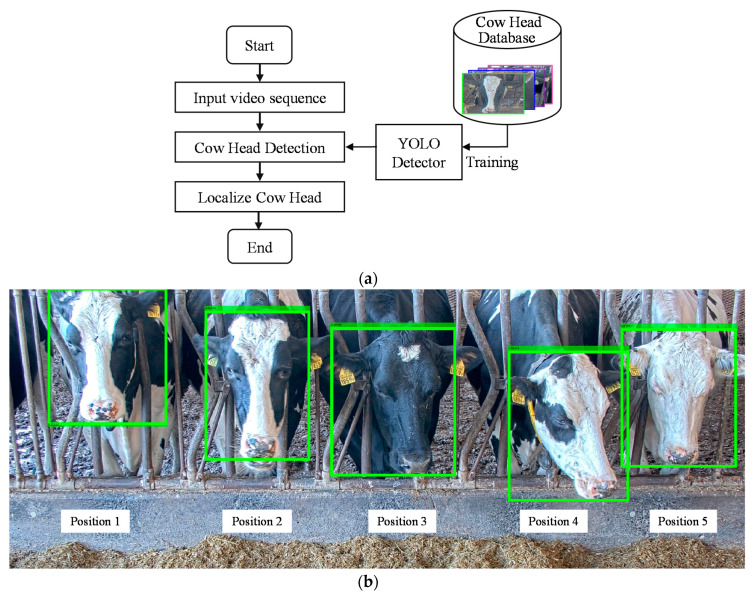
(**a**) Flowchart of cow head detection, (**b**) detected and localized cow heads.

To obtain the ear tag for each cow, we first perform head detection. At the feeding station, 4K network cameras are installed in front of the cows. The feeding station can accommodate a maximum of five cows in each camera view. Only one cow can insert her head through a narrow space between iron bars at the entrance. After head detection, cow localization is performed for each camera view in positions 1, 2, 3, 4, and 5 in that order. To locate the position of the cow, the region between each set of iron bars is pre-allocated to know which cow has entered in the region between which iron bars. The flow for these initial steps can be seen in Figure 3.

Head detection is carried out using a YOLO object detector [18]. This detector is used here because of its speed, which is about 45 frames per second. We prepare the YOLO object detector model as described in [19]. To conduct our experiments, we collected 10,793 image frames from recorded videos (including 44,000 cow heads from these frames). Each image frame can contain the maximum of five cows. From this dataset of 10,793 frames, we used 60% for training, 10% for validation, and 30% for testing. The dataset information is shown in Table 1.

In our network model, we used MobileNetV2 [20] for the feature extraction layer. The Stochastic Gradient Descent with momentum (sgdm) is used for network training and the initial learning rate is set to 0.001 with three epochs.

In the training of a cow head detector, the training image frames with their ground truth bounding boxes are fed into the network. We attained an average precision (AP) of 98% in testing 3238 frames. In the detection of a cow head region, the input is the image frame. The output of applying our trained network is a cow head region, which is marked by the bounding box. This process is shown in Figure 3b.

### 3.2. Ear Tag Detection and Filtering

**Figure 4:**
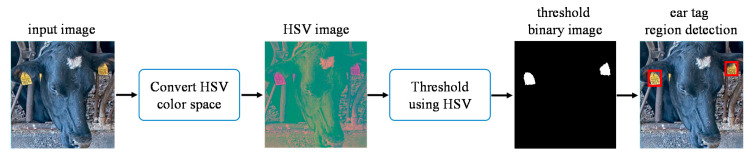
Ear tag region detection from the head image.

After the heads are detected in the input image, ear tag detection is performed. To detect the ear tag region, images returned after the cow head detection are transformed from the RGB (Red, Green, and Blue) to the HSV color space. The three channels of the HSV color space are Hue (H), Saturation (S), and Value (V). By using five videos, the appropriate threshold values for each channel are selected by analyzing all the existing color values that belong to the ear tag region. The selected parameters for the ear tag detection process are described in Equation (1). This process is illustrated in Figure 4.

(1) $$H=\{\begin{array}{cc} 1, & \mathrm{if}\;0.082\leq H\leq 0.263\\ 0, & \mathrm{otherwise} \end{array},\;S=\{\begin{array}{cc} 1, & \mathrm{if}\;0.072\leq S\leq 0.559\\ 0, & \mathrm{otherwise} \end{array}\;\mathrm{and}\;V=\{\begin{array}{cc} 1, & \mathrm{if}\;0\leq V\leq 1\\ 0, & \mathrm{otherwise} \end{array}$$

**Figure 5:**
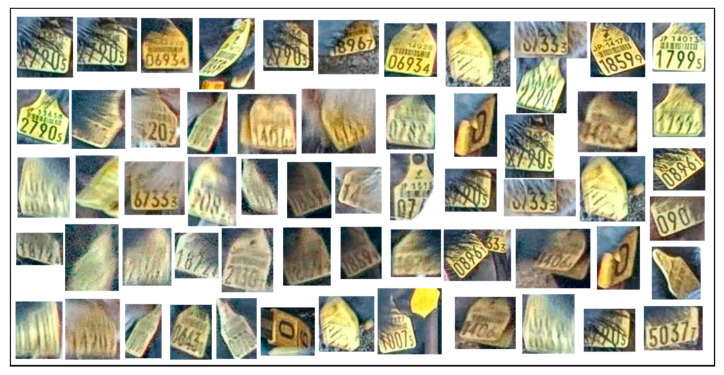
Sample ear tag images after ear tag extraction.

Some noisy images appear when detecting ear tags. These images contain mud and cow feed, which are mistaken for the yellow tags because of their similar color. An example of detected ear tag images is shown in Figure 5. To overcome this problem, we perform two steps of the filtering process on each image.

#### 3.2.1. Initial Noise Removal

In this process, the detected ear tag images are converted into the HSV color space. The threshold parameters are empirically set as described in Equation (2), extracting only ear tag color regions while excluding noisy regions. The segmented binary images are formed after thresholding.

(2) $$H=\{\begin{array}{cc} 1, & \mathrm{if}\;0.128\leq H\leq 0.345\\ 0, & \mathrm{otherwise} \end{array},\;S=\{\begin{array}{cc} 1, & \mathrm{if}\;0.067\leq S\leq 0.582\\ 0, & \mathrm{otherwise} \end{array}\;\mathrm{and}\;V=\{\begin{array}{cc} 1, & \mathrm{if}\;0\leq V\leq 0.667\\ 0, & \mathrm{otherwise} \end{array}$$

We calculate the pixel density values for the ear tag binary image. If the pixel density is between 400 and 2700, we assume this image to be an initial filtered image and save it for the next filtering step. After the initial step of noise removal, other problematic images remain, such as blurred and fur covered images. Such images adversely affect ear tag recognition. Therefore, we remove the blurred and fur covered images in the next filtering step.

#### 3.2.2. Blurred and Fur Covered Image Removal

**Figure 6:**
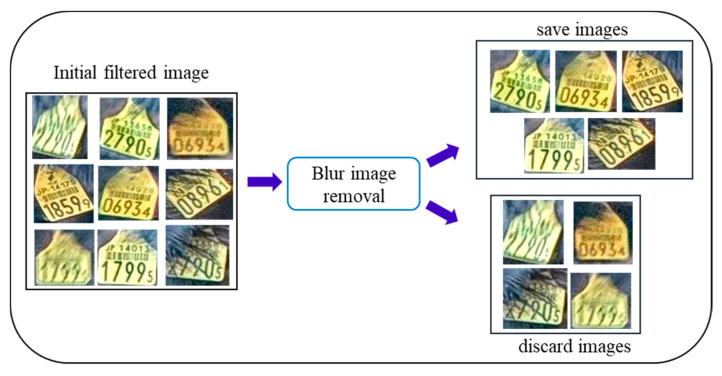
Ear tag filtering.

In the second stage, we extract standard images of the ear tag. To perform the second step filtering, the initial filtered images are resized into 200 × 200. We use the two parameters (contrast and pixel density) to eliminate the fur covered and blurred image. The threshold parameter values are set by using videos analysis results. The threshold range of the standard image used in our work are: The contrast is between 0.17 and 0.65, pixel density is greater than 15,500. This process is shown in Figure 6.

### 3.3. Normalization for Ear Tag Alignment

**Figure 7:**
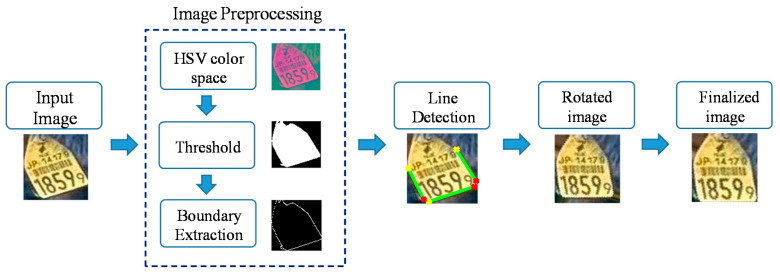
Normalization of ear tag image.

After blurred and noisy images are removed, the next step is image normalization. Due to a variety of positions appearing in ear tag images, some images are skewed or rotated. Therefore, we must perform skew correction on images, and transform them into a horizontal alignment. To detect the horizontal base line for each ear tag image, we use the Hough transform [21] and calculate the incline angle. Finally, the image is rotated into its normal position using the incline angle value. These steps are shown in Figure 7.

Firstly, the input ear tag image is processed using the image preprocessing steps. After we convert the original image into the HSV color space, we threshold the image to focus on the ear tag region. The threshold parameters are the same as those used in the initial noise removal step. After that, we apply the edge detector to find the boundary points of the binary image.

**Figure 8:**
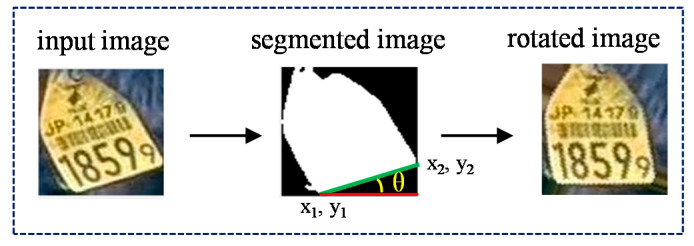
Illustration of skew image correction.

We use the ‘Prewitt’ edge detector [22] in our system and extract the boundary points. After getting results for the binary contour image, the resulting image is inputted into the ‘Hough Transform’ (HT) algorithm to detect the horizontal line and get the base line of the image. The degree of skew can be calculated using a simple linear equation, as described in Equation (2). This process is shown in Figure 8. The normalized ear tag images are then sent to the ear tag recognition stage for further processing.

(3) $$m=\frac{y_{2}-y_{1}}{x_{2}-x_{1}},\;\mathrm{and}\;\theta =\tan^{-1}\left(m\right)$$

where $x_{1}$, $y_{1}$ and $x_{2}$, $y_{2}$ are the X and Y coordinates of a base line, *m* is the slope of a base line, and *θ*: is the degree of a line.

### 3.4. Ear Tag Recognition Process

The goal of ear tag recognition is to identify the digits printed on the ear tag and then establish the position of the individual cow.

#### 3.4.1. Preprocessing

**Figure 9:**
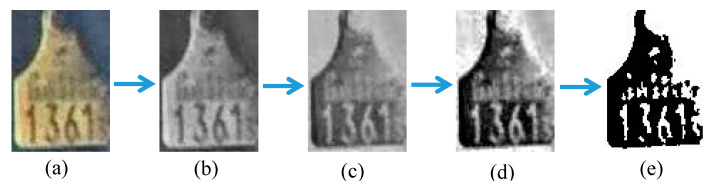
Illustration of step-by-step preprocessing: (**a**) Original Red, Green, and Blue (RGB) image, (**b**) g-scale image, (**c**) inverted image, (**d**) histogram equalized image, and (**e**) binarized image.

Ear tag images obtained from the filtering process must be segmented to get individual digits. To simplify the segmentation step, the ear tag images must pass four preprocessing steps. Some important pixels can be lost in direct binarization. Therefore, the filtered image is first changed into a gray-scale image. This gray image is next inverted as subsequent processes require white digits. Histogram equalization is then performed on the inverted image to get a clear image. Finally, the equalized image is binarized. This process is illustrated in Figure 9.

**Figure 10:**
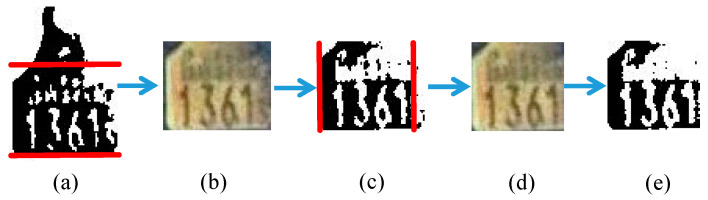
Removal of unnecessary borders: (**a**) Preprocessed image with two horizontal crop lines, (**b**) horizontally cropped original image, (**c**) horizontally cropped preprocessed image with two vertical crop lines, (**d**) unnecessary border removed from original image, and (**e**) preprocessed image with unnecessary borders removed.

In an ear tag image, only areas with digits need to be recognized. Therefore, such areas are extracted by removing other unnecessary borders, as illustrated in Figure 10. The removal steps are as follows:

- Calculate horizontal projection values for the preprocessed image.
- Remove horizontal (upper and lower) borders with projection values that are less than half of the width of the image.
- Crop the original RGB image, preprocess the cropped image, and calculate vertical projection values.
- Then, remove vertical (left and right) borders that have projection values less than two-thirds of the height of the preprocessed image.

#### 3.4.2. Segmentation

The segmentation process is performed according to the type of projection [23]. The types of projection are horizontal or vertical. Horizontal projection is the summation of pixel values for each row; and similarly, vertical projection is the summation for each column.

**Figure 11:**
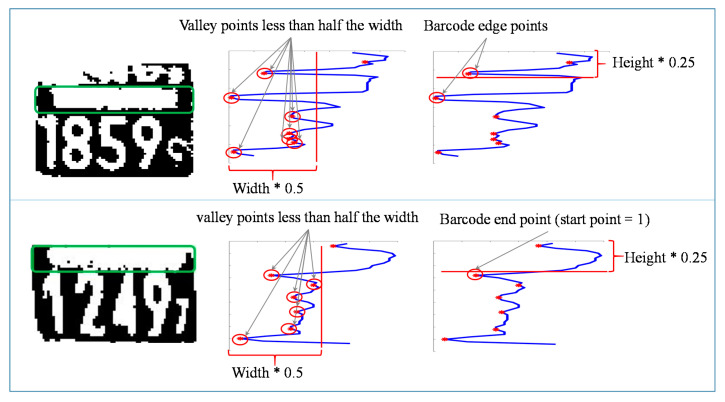
Barcode area detection process.

Horizontal valley points are used in detecting the barcode area. The valley points are horizontal projection values that lie lower than both of their neighbor values. Valley points that are less than half the width of the image are considered predefined barcode end points. Based on empirical results, the barcode is assumed to extend over one-fourth of the image height. Therefore, the first valley point greater than one-fourth of the image height is taken as the barcode end point. If the selected point is the first valley point, then the barcode start point is marked as ‘1’. Otherwise, the prior valley point of the selected barcode end point is taken as the barcode start point. The barcode area detection process is shown in Figure 11.

**Figure 12:**
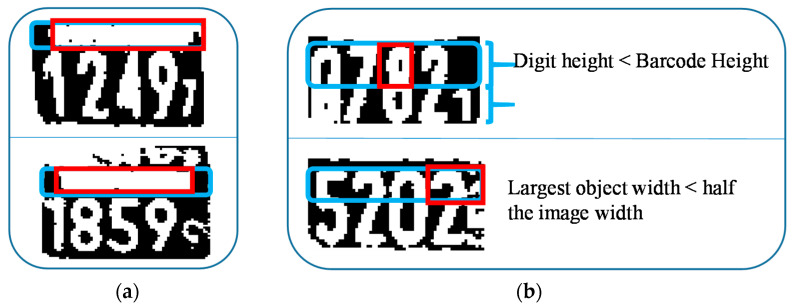
Determination of the digit start point: (**a**) Correctly taken as a start point, (**b**) not taken as a start point.

The barcode end point is taken as the horizontal start point of the digit area if the following two conditions are satisfied. This process is illustrated in Figure 12.

- The remaining digit height must be greater than 1.7 times that of the barcode’s height.
- The largest object width in the barcode area must be greater than half of the image’s width.

**Figure 13:**
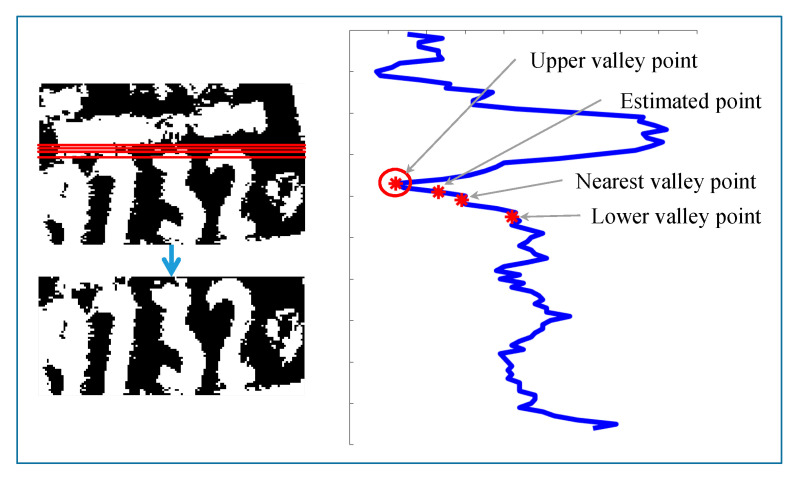
Digit area start point calculation.

Since barcodes are sometimes occluded, barcodes are not detected in some images. For such images, the digit area start point is calculated using a second approach. Firstly, horizontal projection and valley points are calculated. Then, the estimated start point is calculated by multiplying the image width by the threshold value of 0.45, according to empirical results. The projection values for the estimated point, nearest to the valley point, as well as adjacent upper and lower valley points are compared. The minimum projection value among these four points is taken as the digit area start point. This process of calculating the digit area start point is shown in Figure 13.

**Figure 14:**
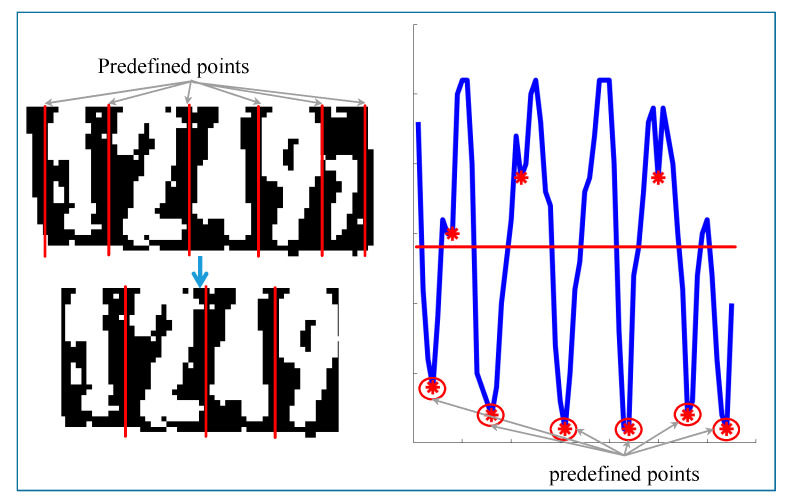
Segmentation of individual digits.

After the digit area is extracted from the original image, individual digits are segmented using vertical valley points. The valley points are the vertical projection values that lie lower than both of their neighbor values. Valley points greater than half the image height are discarded. Since there are four core digits and one mini digit on the ear tag, the minimum possible digit width is assumed to be one-sixth the value of the image width. With the remaining points as well as image horizontal start and end points, the widths between two adjacent points are calculated. The two points for widths greater than the minimum digit width are retained, and other points are discarded. In Figure 14, points 1 through 5 are retained, and the image horizontal start point, end point, and point 6 are discarded. The image is cut into four parts using the resulting five points.

#### 3.4.3. Digit Object Determination

Some segmented parts include more than one digit, or can include mini digits or noise due to the lighting on the image. The digits are distinguished by binarizing the segmented part with the specific threshold value by taking pixel values that are less than 150. From the binarized image, the object that meets the following two criteria are chosen as the digit. The criteria are as follows:

- The object’s width is less than its height; and,
- The object’s height is greater than two-thirds of the image’s height.

**Figure 15:**
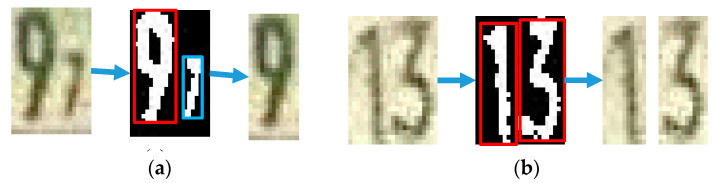
Digit object determination: (**a**) Elimination of mini digits and (**b**) division of closed digits.

If more than one object satisfies these criteria, an average segment line is added between objects. Otherwise, if only one object satisfies these criteria, all other objects are assumed to be noise, and the detected object area is cropped out. In order to preserve the resolution of the final segmented image, each cropping process is performed on an original RGB image. The process of digit object determination is shown in Figure 15.

#### 3.4.4. Ear Tag Recognition

In the system proposed in [24], a convolution neural network (CNN) is applied in the recognition step. The CNN architecture is specified in the first hidden layer, using 16 convolutional filters with a 5 × 5 filter size followed by a batch normalization layer and a rectified linear unit (ReLU) layer. For the second and third hidden layers, we used 32 filters with a 3 × 3 filter size and ReLU layers. Then, we added an average pooling layer with a size of 2 × 2 and stride of 2. After that, we used two consecutive fully connected layers. The output size of the first layer was 100 and that of the second layer was 10, as required to classify 10 digits. The network training and testing accuracy were 96.88% and 94.80%, respectively.

The CNN is trained by using ‘stochastic gradient descent with momentum (sgdm)’ as the solver for the training network, with an initial learning rate of ‘10^−4^’. Currently, a total of ‘10,000’ digits (or ‘1000’ data points for each digit) are used as ‘training’ data; this includes ‘2000’ digits (or ‘200’ data points for each digit) used as ‘testing’ data.

**Figure 16:**
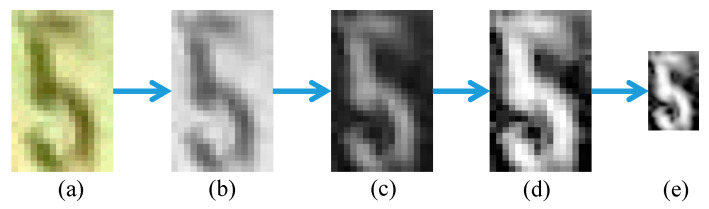
Step-by-step preprocessing of training data: (**a**) Manually cropped RGB image, (**b**) gray-scale image, (**c**) complemented image, (**d**) histogram equalized image, and (**e**) resized image.

In the training process, the individual digits specifically used for training are manually cropped from the video data. The cropped digits are then transformed into gray-scale and complemented. Next, the transformed digits undergo histogram equalization, binarization, and resizing into height and width dimensions of 64 × 32. Each cropped digit is resized into height and width dimensions of 64 × 32. Then, the resized image is classified using the trained CNN. Step-by-step preprocessing for an individual digit is shown in Figure 16.

#### 3.4.5. Ear Tag Confirmation Process

Since the system processes images of actual ear tags, various types of noise occur, such as that caused by variations in lighting, mud, and misplaced fur. The resulting occlusion causes poor accuracy in segmentation. This consequently results in poor recognition accuracy. As mitigation, we perform ground truth data matching to obtain correct information on ear tags, where a matching of four, or even three digits can be useful. As the system includes video processing, the ear tag numbers can be updated on occasion, particularly to cover unrecognized ear tags, or to correct wrongly recognized ear tags.

**Figure 17:**
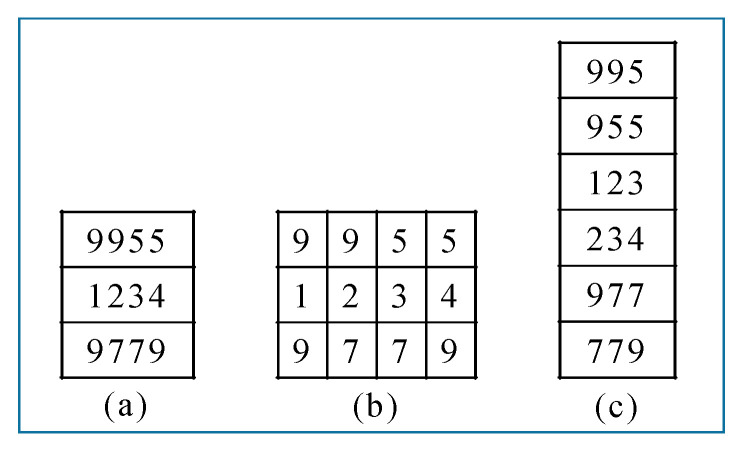
Check lists using the three types of ground truth data: (**a**) Four-digit list, (**b**) one-digit list, and (**c**) three-digit list.

Recognized ear tag numbers are confirmed using three check lists, which are those with four digits, three digits, and one digit. Sample check lists using these three types of ground truth data are shown in Figure 17.

**Figure 18:**
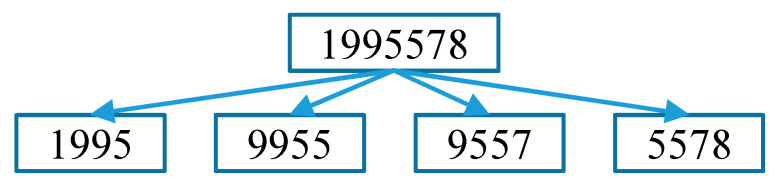
Cutting process for ear tags with length of more than ‘3’.

Ten conditions must be satisfied in deciding whether a recognized ear tag is saved or discarded. We do not consider an ear tag to be recognized when the string length is less than three. If the ear tag length is ‘3’, we check for any similar digit in the three-digit list. For an ear tag with a length of more than ‘3’, we cut into ear tags with a length of ‘4’, as shown in Figure 18.

A search is made for each cut ear tag in the four-digit list. Cut ear tags that do not match with any data in the four-digit list are separated into individual digits. Then, the first digit is checked for any match in the first column of the one-digit list. Similarly, second, third, and fourth digits are checked in their respective columns in the one-digit list. Then, the indices for all possible three-digit pairs are intersected, since we use the number for the ear tag with three correct digits. As an example, ‘124_’, ‘12_9’, ‘1_49’, and ‘_249’ all indicate a correct ear tag number of ‘1249’.

**Figure 19:**
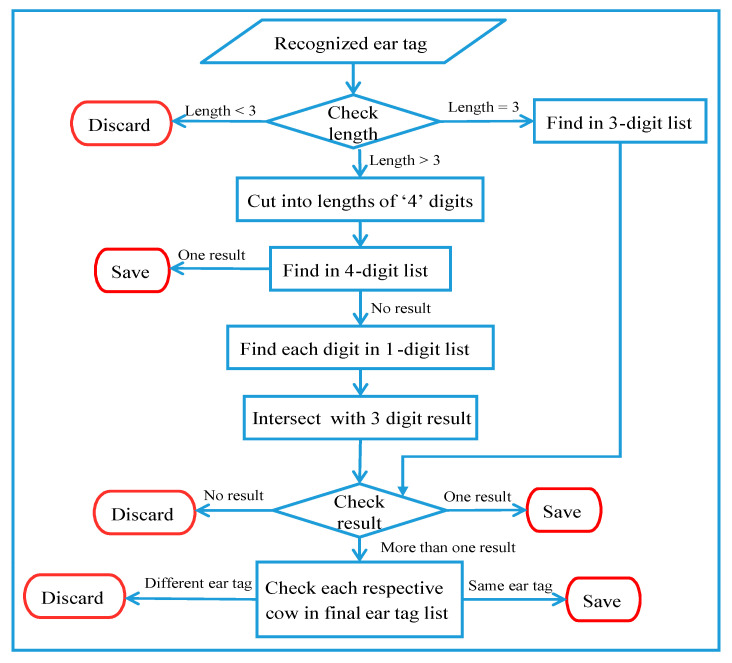
System flow for ear tag confirmation.

A flow chart diagram for the ear tag confirmation process is shown in Figure 19. Once an ear tag is confirmed, it is immediately saved in the initial ear tag list, along with a respective camera number and cow position. The final ear tag list is updated every thirty frames, by choosing the optimal result from the initial ear tag list. For the case in which more than one similar digit is found, a matching with the history ear tag is performed by finding the same ear tag number using the same camera number and cow position as in the final ear tag list.

The recognized ear tag is discarded if it meets one of the following five conditions, described as D1 through D5.

1. The length is less than ‘3’ (D1).
2. The length is exactly ‘3’, but no match is found in the three-digit list (D2).
3. The length is exactly ‘3’ and more than one match occurs in the three-digit list, but no match is found in the ear tag history (D3).
4. No intersected result occurs for any of the three-digit pairs of cut ear tags (D4).
5. More than one intersected result occurs, but no match is found in the ear tag history (D5).

The recognized ear tag is saved if it meets one of the following five conditions, described as S1 through S5.

1. The length is exactly ‘3’ and one match is found in the three-digit list (S1).
2. The length is exactly ‘3’. More than one match is found in the three-digit list and one of the results matches in the ear tag history (S2).
3. One match is found for the cut ear tag in the four-digit list (S3).
4. One intersected result occurs for a three-digit pair of cut ear tags (S4).
5. More than one intersected result occurs, and one match is found in the ear tag history (S5).

The ear tag confirmation process is illustrated in Table 2. The yellow rows represent the recognized ear tags for both left and right sides of four cows. The green rows represent the confirmed ear tags together with their respective constraint numbers. The ‘−’ symbol represents ‘non-recognized ear tag’. The ‘−(D*n*)‘ symbol represents ‘discarded ear tag’, where *n* can be one of five conditions. In the illustration, we consider ground truth data to be ten ear tags of 0004, 0647, 1127, 1246, 1249, 1733, 3140, 5202, 5208, 9230, four cows with correct ear tags of 1127, 0647, 5208, 9230, and three frame sequences.

### 3.5. Decision Making

The frame rate for the network cameras used in our experiments was 25 frames per second, and we only processed one frame to reduce the processing time. Cow head detection and ear tag recognition processes are performed for every input frame. To determine the number of cows and their respective ear tags, we performed a head and ear tag updating process every 30 frames while running the process.

Cows are not always in their regions of interest (ROI) and may sometimes be in another ROI. During such times, both head detection and ear tag recognition results occur in another ROI instead of the actual position. Therefore, we made a decision table using current and previous detection and recognition history. If the occurrence count of each region is greater than 50% of 30 frames, we considered that region to have a cow. This process is illustrated in Table 3. The value ‘1’ indicates the head detection, and ‘0’ indicates that either no detection is made or no head is present. The same process is also performed on the ear tag updating process.

## 4. Experimental Results

The experiments were performed at a large-scale dairy farm in Hokkaido Prefecture, Japan. The network cameras (AXIS P1448-LE) were installed to provide a frontal view of the feeding station. The camera had a resolution of 4K (3840 × 2160) and recorded video at 25 frames per second. One network camera can cover five cows. For data acquisition, the recorded videos are stored in the Network Attached Storage (NAS) every five minutes. Feeding time starts at approximately 7 A.M. In our experiments, videos were randomly selected from the 12th, 13th, and 14th of October, and from the 17th, 18th, 24th, and 25th of November 2019. Each video is five minutes in length, because they are sent from each network camera and stored in NAS every five minutes.

In this video processing, we calculate the cow head detection rate using our decision-making rule (which updates detection results every 30 frames). After recognizing the segmented digits with CNN, we perform the ear tag confirmation process. Then, the final results for ear tag digits are obtained every 30 frames in each respective group of confirmed ear tags. The accuracies for head detection rate and ear tag digit classification for each video are provided in Table 4.

According to experimental results, the proposed system has an accuracy rate of 100% for head detection, and 92.5% for ear tag digit recognition. Some ear tags were not detected due to problems such as noise in the ear tag area, fur covering the tag, and blurred images due to head movement. The proposed system can function in challenging environments, in various and changing weather conditions.

**Figure 20:**
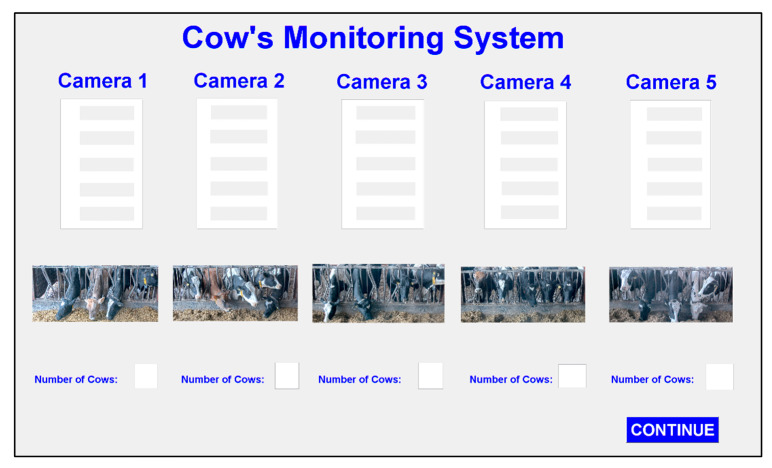
Design of user interface for the proposed system.

The graphical user interface (GUI) of the proposed system is shown in Figure 20. The application accommodates five network camera views. The number of cows involved and their corresponding ear tags are shown in the application.

**Figure 21:**
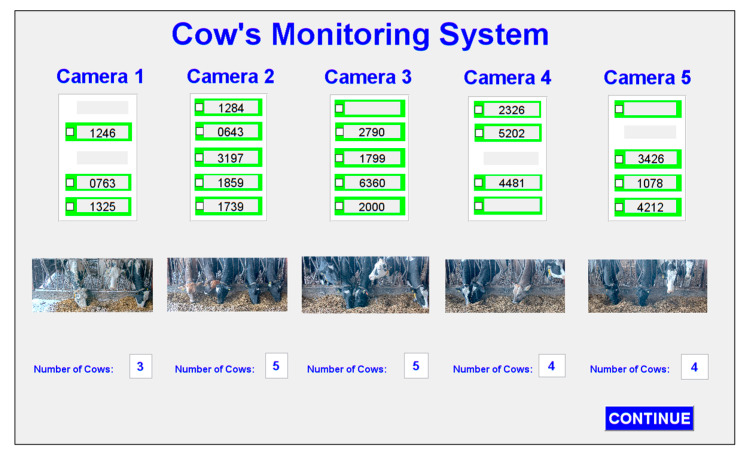
Finished process view of the system.

The result of processing five videos is shown in Figure 21. In this figure, the numbers of cows, corresponding ear tags, and snapshot image views are shown for each camera. If the system can detect a cow, its position is highlighted in green. Recognized ear tag numbers are displayed on the screen and undetected ear tag numbers are left blank. Sometimes, cow head is not in a stable position and they can move to other cow’s region of interest. This can occur duplicate positions of detection.

**Figure 22:**
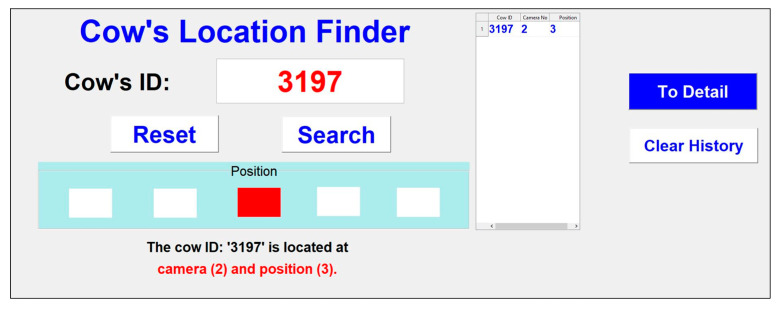
Design for the search system user interface.

When the system completes processing, it is combined with the search system to find another cow. The user needs to enter the ID of the cow to find her actual position. The running time of the system is about 15 min to finish all five videos (5 min each), and processing is performed in parallel. The system was tested using Windows 10, with an Intel^®^ Core (TM) i7-7700 CPU running @ 3.60GHz with a 16 GB memory. The GUI design of the search system is shown in Figure 22.

**Figure 23:**
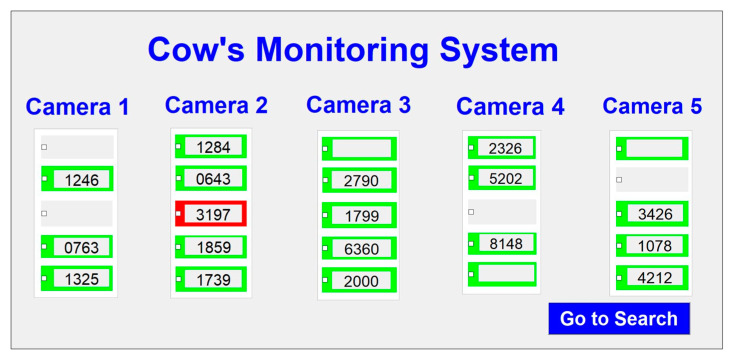
The search system form with detail information.

The search system can identify the camera and its specific position. A detail page is also provided to view the search ID, with its position highlighted in red. This is illustrated in Figure 23.

## 5. Conclusions

Systems for identifying and monitoring individual cows are important in precision dairy farming, because such systems can provide valuable information, such as the status of each cow. Manually searching for cows takes time and increases labor costs. In this paper, we propose a cow identification system using printed ear tag numbers. To extract the ear tag region, we first detect the head area using a pre-trained YOLO detector model. Head detection can also provide the cow’s position, which can then be applied into the cow’s searching system. Step-by-step processes are carried out to extract the digit area and ear tag recognition is then performed. The proposed system has already been tested in the real-world environment of a working dairy farm. The cow finding system also provides information on the desired location for each cow. According to experimental results, the proposed system shows promise as a reliable and useful contribution to smart dairy farming. To have a better recognition accuracy, we need to make modifications in the ear tag detection process for better extraction of the ear tag’s digit, which can improve the recognition accuracy. For the future, we will extend our work to apply in different livestock environments.

## Figures and Tables

**Table 1:** The dataset information.

Dataset	#Image Frames	#Cow Heads
Training	6475	26,371
Validation	1080	4429
Testing	3238	13,227

**Table 2:** Brief illustration of the ear tag confirmation process.

Ear Tag Confirmation	Cow 1		Cow 2		Cow 3		Cow 4	
	Left	Right	Left	Right	Left	Right	Left	Right
**Frame 1**	112	-	069	064	5208	52087	99231	7231
**Result 1**	1127(S1)	−(D1)	−(D2)	0647(S1)	5208(S3)	5208(S3)	9230(S4)	−(D4)
**Frame 2**	11	1127	11641	064	520	15203	9230	9230
**Result 2**	−(D1)	1127(S3)	−(D4)	0647(S1)	5208(S2)	5208(S5)	9230(S3)	9230(S3)
**Frame 3**	124	1247	0647	11647	5208	5202	7280	9230
**Result 3**	−(D3)	−(D5)	0647(S3)	0647(S4)	5208(S3)	5202(S3)	−(D4)	9230(S3)

**Table 3:** Illustration of the cow head decision table.

Frames	Cow Head Regions				
	ROI 1	ROI 2	ROI 3	ROI 4	ROI 5
1	1	0	1	0	1
2	1	1	1	0	1
3	1	0	1	0	1
…	…	…	…	…	…
29	1	0	1	1	0
30	1	0	1	0	1
Occurrence Count	27	5	30	3	27
Percentage	90%	16%	100%	10%	90%
Cow Present	‘Yes’	‘No’	‘Yes’	‘No’	‘Yes’

**Table 4:** Experimental results for head detection and ear tag digit classification.

No.	Video	Number of Cows	Head Detection Rate	Ear Tag Digit Classification Rate
1.	Video 1	5	100%	100% (5 out of 5)
2.	Video 2	5	100%	80% (4 out of 5)
3.	Video 3	4	100%	100% (4 out of 4)
4.	Video 4	4	100%	100% (4 out of 4)
5.	Video 5	4	100%	100% (4 out of 4)
6.	Video 6	5	100%	100% (5 out of 5)
7.	Video 7	5	100%	100% (5 out of 5)
8.	Video 8	5	100%	80% (4 out of 5)
9.	Video 9	5	100%	100% (5 out of 5)
10.	Video 10	5	100%	80% (4 out of 5)
11.	Video 11	3	100%	100% (3 out of 3)
12.	Video 12	5	100%	100% (5 out of 5)
13.	Video 13	5	100%	80% (4 out of 5)
14.	Video 14	4	100%	75% (3 out of 4)
15.	Video 15	4	100%	75% (3 out of 4)
16.	Video 16	5	100%	80% (4 out of 5)
17.	Video 17	4	100%	100% (4 out of 4)
18.	Video 18	3	100%	100% (3 out of 3)
19.	Video 19	5	100%	100% (5 out of 5)
20.	Video 20	5	100%	100% (5 out of 5)
Overall Accuracy			100%	92.5%
